# Editorial: Epigenetic and metabolic regulation of immunity during infection or cancer and associated immune biomarkers

**DOI:** 10.3389/fimmu.2023.1275735

**Published:** 2023-08-22

**Authors:** Valeria Barili, Paola Fisicaro, Carolina Boni

**Affiliations:** ^1^ Medical Genetics, Department of Medicine and Surgery, University of Parma, Parma, Italy; ^2^ Laboratory of Viral Immunopathology, Unit of Infectious Diseases and Hepatology, Azienda Ospedaliero-Universitaria di Parma, Parma, Italy

**Keywords:** gene expression, metabolism, immunity regulation, infection, cancer

The intricate interplay between epigenetic modifications, metabolic processes, and immune responses has garnered significant awareness in the context of infections and cancer (1, 2). Epigenetic changes, such as DNA methylation and histone modifications, can dynamically shape immune cell differentiation, function, and memory formation during infection and malignancies (3–5). Simultaneously, metabolic pathways govern energy production, biosynthesis, and immune cell fate determination, finely orchestrating immune responses. The bidirectional influence of epigenetic modifications and metabolic pathways underscores their pivotal roles in modulating immune cell behaviour in disease contexts (6, 7).

Fundamental is to delve into the impact of epigenetic alterations on immune cell differentiation, polarization, and memory formation, highlighting their implications for pathogen clearance and antitumor immunity. Moreover, exploring how metabolic reprogramming shapes immune cell phenotypes, from effector functions to immune checkpoint regulation, fosters a deeper understanding of immune evasion mechanisms in cancer and infections. Furthermore, epigenetic modifications and metabolic signatures can serve as valuable indicators of disease states, treatment responses, and patient outcomes outlining the promise of personalised medicine approaches guided by epigenetic and metabolic profiles.

Thus, the definition of reliable biomarkers able to predict patients’ response to treatment, including immunotherapeutic strategies, represents a pivotal goal in order to improve and personalise the cure for malignancies, as well as the discovery of prognosis-associated prediction factors.

To this end, unravelling publicly available datasets by thorough bioinformatic analysis, the study by Tong and Zhou establishes a score based on the expression level of genes related to mitochondrial metabolism (MMRGs), able to identify two Acute Myeloid Leukaemia (AML) patients groups (defined “high” and “low-risk” respectively), with significantly different survival outcomes. Starting from the analysis of 31 MMRGs and the patients’ survival information the authors could select a signature of 5 genes differentiating high and low-risk patients. Notably, immunohistochemistry staining of bone marrow biopsies validated the higher expression of some of these mitochondrial genes in AML compared to non-neoplastic patients. Also literature-selected DNA repair genes resulted up-regulated in high risk patients, underlining the association between dysregulated metabolism and genomic instability. Interestingly, genes differentially expressed between high and low risk patient groups resulted mainly involved in immune response regulation, mitochondrial metabolism and drug-resistance pathways, highlighting not only a link between drug resistance and mitochondrial metabolism, but also the higher probability for immune dysfunction in the high-risk patient group, that can thus be expected to respond less efficiently to an immunotherapeutic intervention.

In addition, the article by Gou et al., while focused on the immuno-pathogenetic study of digestive system cancers (DSCs), and in particular hepatocellular carcinoma (HCC), ends up identifying a potential diagnostic biomarker. Due to the involvement of Zinc in several biological processes and the documented presence of low Zinc transporter proteins (ZIPs) levels in different human cancers, this study explores the intrahepatic gene expression of ZIPs, emphasising the relevance of their alteration in the HCC model. Indeed, several ZIPs, including ZIP2 and ZIP9, resulted to be down-regulated within the liver of HCC patients, as well as in a HCC mouse model, and some of them were shown to be linked to macrophage polarization. Particularly, through different *in vitro* and *in vivo* experiments, including ZIP9 knock-down, the authors could observe inhibition of M2 development, due to the suppression of STAT6 phosphorylation, while M1 macrophage differentiation was promoted through IkBα/β phosphorylation. Given the relevance of the role of macrophages in the development of the inflammatory environment and in the evolution of tumorigenesis, these findings not only shed light on the HCC pathogenesis, but also suggest ZIP9 down-regulation as a potential marker associated to HCC development.

Yuan et al.‘s study highlights the pivotal role of Glucosamine 6-phosphate N-acetyltransferase (GNPNAT1) in breast cancer (BRCA) progression and its diagnostic and prognostic implications. GNPNAT1, a key enzyme in the hexosamine biosynthesis pathway (HBP), exhibits significant up-regulation in multiple cancer types, particularly BRCA. The research consistently demonstrates elevated GNPNAT1 expression in BRCA tissues, correlating with clinical characteristics and overall survival. Functional analysis links GNPNAT1 to BRCA hallmarks, related to proliferation and invasion. GNPNAT1’s diagnostic potential is evident through ROC curves, showing promise in distinguishing cancer from normal tissues and cancer stages. Its prognostic significance is validated by survival analysis, particularly in specific BRCA subtypes. The study identifies differentially expressed genes linked to GNPNAT1, uncovering potential mechanisms driving its effects, including metabolism and DNA repair processes. Immune-related processes are enriched in the low GNPNAT1 expression group, connecting its role in immune modulation. The study also reveals GNPNAT1’s impact on the immune microenvironment and its potential adverse effect on immune checkpoint inhibitor efficacy in triple-negative breast cancer (TNBC) patients. The paper establishes GNPNAT1 as a significant player in breast cancer progression and highlights its potential as a diagnostic and prognostic biomarker. The findings underscore its complex role in influencing tumour growth, invasion, immune microenvironment, and response to immunotherapy, particularly in TNBC. The insights gained from this research provide a foundation for further studies and potential clinical applications in breast cancer management.

Moreover, Liang et al.‘s study employs bioinformatics analysis and Raman spectroscopy to differentiate between acute myeloid leukaemia (AML) and T cell acute lymphoblastic leukaemia (T-ALL). The research explores gene expression profiles, identifying differentially expressed genes (DEGs) related to immune response, inflammation, and signaling pathways. This comprehensive analysis not only confirms the genetic basis of AML and T-ALL, but also sheds light on the distinct biological processes underlying these diseases. To further elucidate the relationships and interactions between the identified DEGs, a protein-protein interaction (PPI) network is constructed, highlighting key genes like TLR4 and CSF1R distinguishing AML from T-ALL. A notable aspect of this study is the integration of Raman spectroscopy, a non-invasive analytical technique that provides insights into the molecular composition of samples. By analysing Raman spectra of bone marrow samples from AML and T-ALL patients, the study aims to validate the previously identified biomarkers and assess their potential as diagnostic indicators. The results indicate that certain Raman peaks correspond to distinctive molecules, which can be used to discriminate between AML and T-ALL samples. Thus, these findings not only enhance our understanding of the molecular bases of these two leukaemia types but also hold promising implications for enhanced diagnostics and personalised treatment approaches in the future.

Finally, adoptive immunotherapy with multi-pathogen-specific T cells as a novel cell therapy tool for the treatment of opportunistic infections in transplanted patients has been addressed in the study by Koukoulias et al. As a promising alternative to drug treatment, the authors generated a steroid-resistant T-cell product simultaneously targeting four viruses (CMV, EBV, AdV, BKV) and the fungus Aspergillus fumigatus by genetic disruption of the glucocorticoid receptor (GR) gene using CRISP/CAS9 editing. Immunosuppressive drugs significantly impair T-cell functionality confining the use of antigen-specific T cells only to patients in whom immunosuppression has been withdrawn. This strategy overcomes this limitation by combining the generation of pathogen-specific T cells and concurrent glucocorticoid-resistance by the genetic inactivation of the GR. Adoptive immunotherapy with virus-specific T cells could considerably reduce the transplant-related mortality associated with opportunistic infections and ultimately improve the outcome of immune-compromised patients after allogeneic hematopoietic cell transplantation or solid organ transplantation.

In conclusion, unravelling the intricate web of epigenetic and metabolic regulation of immunity holds profound implications for advancing our understanding of infections and cancer. The identification of immune biomarkers driven by these mechanisms opens avenues for novel therapeutic strategies and precision medicine interventions, ultimately enhancing our ability to combat diseases and tailor treatments to individual patients.

## Author contributions

VB: Writing – original draft, Writing – review & editing. PF: Writing – original draft, Writing – review & editing. CB: Writing – original draft, Writing – review & editing.
